# The Role of Dispositional Orientations and Goal Motives on Athletes’ Well- and Ill-Being

**DOI:** 10.3390/ijerph19010289

**Published:** 2021-12-28

**Authors:** Natalia Martínez-González, Francisco L. Atienza, Joan L. Duda, Isabel Balaguer

**Affiliations:** 1 Department of Social Psychology, Faculty of Psychology and Speech Therapy, University of Valencia, 46010 Valencia, Spain; isabel.balaguer@uv.es; 2 Department of Personality, Evaluation and Psychological Treatment, Faculty of Psychology and Speech Therapy, University of Valencia, 46010 Valencia, Spain; francisco.l.atienza@uv.es; 3 School of Sport, Exercise and Rehabilitation Sciences, University of Birmingham, Birmingham B15 2TT, UK; j.l.duda@bham.ac.uk

**Keywords:** goal orientation, goal motives, sport, vitality, exhaustion

## Abstract

Findings in different contexts suggest that task orientation and ego orientation are related to adaptive and maladaptive motivational patterns, respectively. In sport, these personal dispositions could influence other important variables such as the goals that athletes pursue (and why they pursue them) during the season and their well- and ill-being. The main purpose of this research was to examine the relationship between athletes’ dispositional goal orientations, their goal motives, and their reported well-being (subjective vitality) and ill-being (physical and emotional exhaustion). The study involved 414 Spanish university athletes (206 female and 208 male) with an age range of 17 to 33 years (*M* = 20.61; *SD* = 2.58) that completed a package of questionnaires at the beginning of the season. Results of path analysis revealed that athletes’ task orientation was negatively associated to physical and emotional exhaustion indirectly through autonomous and controlled goal motives. In contrast, ego orientation was positively related to physical and emotional exhaustion via its link to controlled goal motives. Athletes’ task orientation directly and positively predicted subjective vitality, even though goal motives were not significant mediators. These findings support previous evidence about the protective role of athletes’ task orientation, in contrast to ego orientation, confirming its positive relationship with well-being and its negative one with ill-being. Additionally, it extends the knowledge regarding interdependencies between goal orientations and goal motives and how both contribute to athletes’ optimal or compromised functioning.

## 1. Introduction

Over recent decades, many studies have tried to explain why not all athletes benefit from participating in sport. Despite the fact that practicing sport is considered to be an inherently rewarding activity that contributes to people’s well-being [1], sometimes sport participants experience negative psychological and physical consequences [2,3]. In order to explain these differential experiences and outcomes, various social and personal factors that facilitate or hinder the quality of athletes’ sports involvement have been examined in past research [4,5,6,7]. One of the personal factors that is of great importance in competitive sporting contexts is the way in which athletes tend to define success, or differences in dispositional goal orientations. Goal orientations can be predictors, not only of the quality of sport participation, but also of differences in why we pursue the goals that direct the behavior and responses of athletes over the season [8]. Thus, dispositional differences in how athletes judge success can differentially influence the personal experience of their sport participation, even to the point of optimizing or compromising their functioning.

According to achievement goal theory (AGT) [8,9], individuals differ in the manner of how they judge their competence and, therefore, in what criteria they tend to use to realize personal success. In this way, this theory distinguishes two types of dispositional goal orientations depending on the criteria used to define success: task and ego orientation. Highly and predominantly task-oriented athletes base their perceptions of competence on self-referenced criteria and, as a result, they feel competent when they exert effort, do their best, learn, and gain task-mastery. Strongly ego-oriented athletes judge their competence based on normative-based criteria, so they experience feelings of success when they perform better or equal than others but with less effort. Past sport research has found task orientation to positively predict indicators of more satisfying and committed sport participation than ego orientation [10]. Previous evidence suggests that task orientation and ego orientation are related with adaptive and maladaptive motivational patterns and indices of well- and ill-being, respectively [11,12].

One assumption embedded in achievement goal theory is that self-referenced criteria for judging competence/experiencing success is more controllable in comparison with normative-based criteria, because the latter depends on others’ performance and thus tends to be out of the individual’s control [3]. Hence, highly task-oriented athletes are more likely to feel that they have control of one’s own actions, whereas for strongly ego-oriented individuals, the control is more alien to them [3,6]. Past research has offered support to these theoretical assumptions confirming that task orientation is positively related to processes and perspectives that emanate from the self, in contrast with ego orientation, which is guided by and linked to factors that are more external. There is considerable evidence regarding the positive relationship between task orientation and autonomous forms of motivation [13,14,15,16,17,18,19,20], whereas ego orientation tends to relate with less self-determined or controlled motivation [17,18,19,20,21,22]. However, less is known about the relationship between dispositional orientations and other variables, such as motivation regulations, that can be more or less concordant with the self, i.e., the motives for the goals that people pursue.

Grounded within the self-determination theory (SDT) [23,24] framework, the self-concordance model (SCM) [25] places emphasis on the degree to which personal goals self-generated by people (idiographic goals) reflect their inherent values and interests. In this way, these personal goals could be autonomous or controlled, as defined within SDT, in function of if they are more or less concordant with the self. Autonomous goal motives are aligned with individuals’ personal interests and values, they can provide enjoyment and interest, and they tend to be perceived as important by the person. Controlled goal motives are regulated by external (e.g., to obtain rewards or social approval) or internal (e.g., to avoid unpleasant emotions such as guilt) pressures.

Studies that have to date linked AGT and SCM have focused on analyzing the reasons underlying the achievement goals, in a perspective that detaches reasons (the “why”) from aims (the “what”). In this manner, this perspective within the AGT postulates that reasons why individuals pursue achievement goals can predict significant variance in affect-based (i.e., well-being) and behavioral (i.e., sportspersonship) outcomes [26,27]. Past research in sport, for example, has shown that the negative relationship between mastery-approach goals and negative affect was only significant for those athletes who were pursuing the goals with high levels of controlled motivation [28]. However, in these studies, participants were asked to evaluate the degree to which they pursued the predefined achievement goals for reasons that were more or less self-concordant. In contrast, there are no studies that have explored the relationship between athletes’ dispositional orientations with the motivation that underlie athletes’ self-generated goals, i.e., in relation to the motives for pursuing the goals that athletes set for themselves (personal goals). Given the above discussion of the relationship between dispositional orientations and the degree to which these are differentially related to variables that emanate more or less from the self, it could be assumed that a highly task-oriented person would pursue his or her self-generated goals for more autonomous and self-related reasons, whereas a primarily ego-oriented person would do so for more controlled reasons. However, these predicted relationships have not yet been explored in previous research.

Regarding the implications of pursuing goals with more or less concordant motives, one of the main assumptions of SCM is that individuals who strive to meet their personal goals because of autonomous goal motives will exhibit higher well-being and less ill-being [25,29,30]. This could be explained by the point that the sense of volition, agency, and empowerment captured in autonomous goal motives would be expected to correspond to a decreased experience of emotional distress and ill-being [25,31]. In sport, it has been shown that autonomous goal motives are positively related to well-being indicators such as a composite well-being index [32,33] and subjective vitality [34,35,36]. Autonomous goal motives are negatively related with ill-being indicators such as burnout and physical ill-being symptoms [34]. Conversely, controlled goal motives have been positively related to athletes’ ill-being [28] and negatively related to reported well-being [28,32].

To date, there are no studies that have jointly analyzed the relationships between dispositional goal orientations, individuals’ underlying motivation for their goal striving, and reported well- and ill-being. Knowing the way in which athletes judge the success and its relationship with their goal motives and their optimal functioning, could contribute to a better understanding of the individual differences in sport contexts, with all the practical implications that this entails (e.g., in well-being promotion programs). Drawing from the relevant theoretical frameworks (AGT, SCM) and past research, the overarching aim of the present study is to determine the relationship between dispositional orientations (task, ego), goal motives (autonomous, controlled), and athletes’ well- and ill-being (captured via their reported subjective vitality and physical and emotional exhaustion, respectively).

In order to meet this objective, the following hypotheses were tested: (1) task orientation would be positively related to autonomous goal motives and negatively related to controlled goal motives; (2) ego orientation would be positively related to controlled goal motives and negatively related to autonomous goal motives; (3) autonomous goal motives would be positively related to subjective vitality and negatively related to physical and emotional exhaustion; (4) controlled goal motives would be positively related to physical and emotional exhaustion and negatively related to subjective vitality; and (5) goal motives would act as mediators in the relationships between goal orientations and well- and ill-being.

## 2. Materials and Methods

### 2.1. Participants

The study involved 414 athletes with an age range of 17 to 33 years (*M*_age_ = 20.61; *SD* = 2.58), belonging to different university teams from Valencia (Spain). A total of 206 female and 208 male university students who competed in the annual Autonomic Championship of University Sports (CADU) participated in the research. Represented in the sample were athletes who practiced basketball (*n* = 75), handball (*n* = 61), football (*n* = 81), indoor football (*n* = 68), rugby (*n* = 64), and volleyball (*n* = 65).

### 2.2. Procedure

The study was evaluated and approved in the first instance by the Human Research Ethics Committee of the university. Subsequently, researchers presented the project to the directors of sport services and requested participation in it. After obtaining approval from the directors, coaches were informed about the study objectives and the days to collect the data were established. Before the data collection, the questionnaires were adapted to male and female, in order to comply with the demands of Spanish language. At the beginning of the season, trained researcher members administered the questionnaire pack at the training field, after explaining to athletes the confidentiality and anonymity nature of their participation and obtaining their informed consent.

### 2.3. Measures

#### 2.3.1. Dispositional Orientations

Goal orientations were assessed through the Spanish version [37] of the Task and Ego Orientation in Sport Questionnaire (TEOSQ) [38,39]. The questionnaire contains 13 items of which 7 evaluate task orientation (e.g., “I feel successful in sport when I work really hard”) and 6 evaluate ego orientation (e.g., “I feel successful in sport when the others can’t do as well as me”). Responses are endorsed using a 5-point Likert scale from 1 (*strongly disagree*) to 5 (*strongly agree*). Previous studies with Spanish athletes have obtained evidence of satisfactory levels of reliability (α = 0.76 to 0.88) and validity of this scale [17,40,41].

#### 2.3.2. Personal Goal Motives

Self-concordance of goals was measured through the idiographic methodology proposed by Sheldon and Elliot [25], recently adapted to the Spanish sport domain by Martínez-González and colleagues [35]. In this regard, athletes identified the most important sporting goal that they hoped to make progress on during the current season and rated the extent to which they were striving toward this goal for autonomous (4 items, e.g., “Because of the fun and enjoyment the goal provides me”) or controlled (4 items, e.g., “Because someone else wants me to”) motives. Responses were given on 7-point Likert scale ranging from 1 (*not at all*) to 7 (*very much so*). Initial reports using the Spanish validation have shown acceptable reliability (α = 0.67 to 0.70) and validity [36,42].

#### 2.3.3. Subjective Vitality

Athletes’ subjective vitality was assessed through the Spanish version [43] of the Subjective Vitality Scale (SVS) [1]. This version evaluates the subjective experience of being fully of energy and alive using 6 items (e.g., “I feel alive and vital”). All responses range on a 7-point Likert scale from 1 (*not at all true*) to 7 (*very true*). The instrument has shown high internal consistency (α = 0.77 to 0.89) and evidence of good validity in Spanish samples of athletes [4,35,36,44,45,46].

#### 2.3.4. Physical and Emotional Exhaustion

A subscale from the Spanish version [47] of the Athlete Burnout Questionnaire (ABQ) [48] was used to assess athletes’ physical and emotional exhaustion. It used 5 items (e.g., “I am exhausted by the mental and physical demands of sport”) rated by a Likert scale from 1 (*almost never*) to 5 (*almost always*). This subscale of burnout has been used in previous research in the sport domain, showing adequate reliability (α = 0.76 to 0.88) and validity [49,50,51,52].

### 2.4. Statistical Analyses

**Figure 1:**
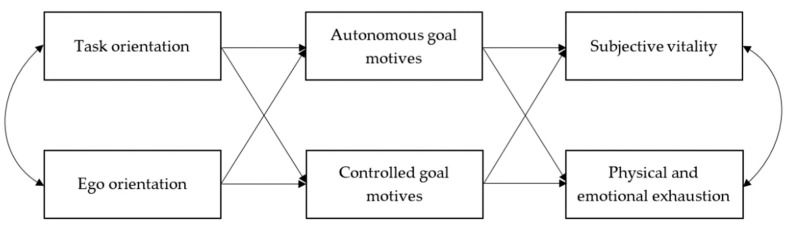
Hypothesized path model of the associations between task and ego orientation, autonomous and controlled goal motives, subjective vitality, and physical and emotional exhaustion.

Descriptive statistics, reliabilities, and bivariate correlations were carried out with IBM SPSS Statistics 25 software (IBM Corporation, Armonk, NY, USA). After prior analyses, a hypothesized model was tested that considered the goal motives as mediators between the athletes’ dispositional orientations and their well- and ill-being (see Figure 1). Due to the number of parameters, mean scores were employed as indicators of the targeted variables, and path analysis was conducted using Mplus (Version 7) [53]. As fit indices, chi-square (χ^2^), the comparative fit index (CFI), the Tucker-Lewis index (TLI), the root mean square error of approximation (RMSEA), and the standardized root mean square residual (SRMR) index were used. For CFI and TLI, values higher than 0.90 indicate an acceptable fit [54]. Values of RMSEA and SRMR between 0.05 and 0.10 are considered acceptable, equal to or lower than 0.08 is optimal [55]. Moreover, bias-corrected (BC) bootstrap 95% confidence intervals (Cis) were applied to test the significance of indirect effects.

## 3. Results

### 3.1. Preliminary Analyses, Descriptive Statistics, and Reliabilities

Preliminary analyses were carried out in order to analyze missing data and outliers. As all variables omitted values below 5%, the criterion for accepting missing data was met. Regarding outliers, the Mahalanobis distance was used to screen for multivariate outliers [56,57]. Three participants were identified as outliers and were removed, leaving a final sample of 411 participants.

Results of the descriptive analyses and scale reliability coefficients are presented in Table 1. The mean scores indicated that athletes were high in task orientation and autonomous goal motives, low in ego orientation and low in controlled goal motives. Regarding well- and ill-being, athletes reported having a high subjective vitality and low physical and emotional exhaustion.

Cronbach’s alpha values showed satisfactory reliability for all the scales (between 0.70 and 0.89), except for autonomous goal motives, which was marginal (0.67). In addition, skewness, and kurtosis coefficients were in the acceptable range of (−1.5, 1.5) for normal distributions [58].

### 3.2. Bivariate Correlations

Correlation analysis showed that the variables correlate significantly in the expected direction, except for the relationship between controlled goal motives and subjective vitality, which was not significant (see Table 2). Specifically, task orientation was significantly and positively correlated with autonomous goal motives and subjective vitality, and negatively related to controlled goal motives and physical and emotional exhaustion. Ego orientation was only significant and positively correlated with controlled goal motives. Additionally, autonomous goal motives were positively correlated with subjective vitality and negatively with physical and emotional exhaustion, whereas controlled goal motives were only negatively correlated with physical and emotional exhaustion. In accordance with the theory, task orientation was negatively related to ego orientation, as well as autonomous with controlled goal motives, and the indicator of well-being with the indicator of ill-being. Of all the significant relationships, the strongest correlation was between task orientation and autonomous goal motives.

### 3.3. Path Analysis

**Figure 2:**
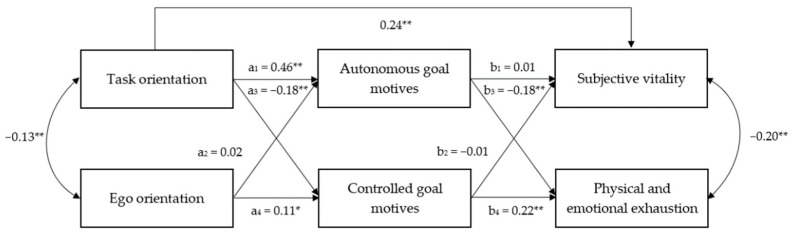
Path model of the associations between task and ego orientation, goal motives, subjective vitality, and physical and emotional exhaustion. * *p* < 0.05, ** *p* < 0.01.

The hypothesized model did not adequately fit to the data (χ^2^ (5) = 29.45, *p* = 0.001, RMSEA = 0.109, CFI = 0.88, TLI = 0.66, SRMR = 0.046). The modification indices indicated adding a direct path from task orientation to subjective vitality. Taking into account the AGT assumptions, it was considered that there were sufficient theoretical reasons to add this path. After this adjustment, the model fitted the data well (χ^2^ (4) = 10.32, *p* = 0.035, RMSEA = 0.060, CFI = 0.97, TLI = 0.90, SRMR = 0.027). The parameters of the standardized solution are displayed in Figure 2. As expected, task orientation was a positive predictor of autonomous goal motives and a negative predictor of controlled goal motives. In contrast, ego orientation only predicted positively controlled goal motives. Regarding well-being, after adding the direct path between task orientation and subjective vitality, the path between autonomous goal motives and subjective vitality was no longer significant. This is to say, in the adjusted model, only task orientation was a significant predictor of athletes’ subjective vitality. Finally, physical and emotional exhaustion were negatively predicted by autonomous goal motives and positively by controlled goal motives.

Both the indirect effects of task orientation on physical and emotional exhaustion via autonomous and via controlled goal motives were significant (IE_a1b3_ = −0.15, BC bootstrap 95% CI = [−0.29, −0.04] and IE_a3b4_ = −0.08, BC bootstrap 95% CI = [−0.16, −0.02]). Moreover, the indirect effect of ego orientation on physical and emotional exhaustion through controlled goal motives was significant (IE_a4b4_ = 0.02, BC bootstrap 95% CI = [0.01, 0.06]). Results of the proposed model significantly predicted 21% of the variance in autonomous goal motives, 5% of the variance of controlled goal motives, 6% of the variance of the reported subjective vitality, and 9% of the variance of the reported physical and emotional exhaustion.

## 4. Discussion

Based on AGT [8,9] and the SCM [25], the aim of the present study was to analyze the relationships between athletes’ dispositional orientations, their goal motives, and their well- and ill-being. The possible role of autonomous and controlled goal motives as mediators in the relationship between task and ego orientation and athletes’ subjective vitality and physical and emotional exhaustion were also tested. A hypothesized model was tested via path analysis and the results revealed partial support for the model.

The first hypothesis, which postulated that task orientation would be positively related to autonomous goal motives and negatively related to controlled goal motives, was confirmed. The results were consistent with the assumption that task-oriented athletes pursue goals that are more concordant with them because they feel more in control of their actions and are more geared toward self-referenced processes [3,6]. Past sport studies have provided evidence in this regard, showing that task orientation has a positive relationship with variables emanating from the self, such as self-determined or autonomous forms of motivation [13,14,15,16,17,18,19,20].

Regarding the second hypothesis, the results supported the positive relationship between ego orientation and controlled goal motives. However, there was no significant relationship between this personal disposition and autonomous goal motives. These findings provided evidence that highly ego-oriented athletes pursue goals for reasons that are more outside their personal interests and values, which is in accordance with previous findings that have reported positive associations between ego orientation and less self-determined or controlled motivation regulations [17,18,19,20,21,22].

With respect to the third hypothesis, autonomous goal motives were negatively related to physical and emotional exhaustion, but (contrary to our hypothesis) were not significantly associated to subjective vitality. Path analysis confirmed that autonomous goal motives were negatively associated with physical and emotional exhaustion [34]. To explicate this unexpected finding, we need to take into account all the analyses conducted and the model tested. Bivariate correlations indicated that autonomous goal motives and subjective vitality were positively but slightly related. Our first model tested with path analysis suggested a positive relationship between these variables. However, this model did not fit well, and modification indices suggested adding a direct path from task orientation to subjective vitality, and thus we obtained the final (well fitting) model. After adding the direct path, the relationship between autonomous goal motives and subjective vitality ceased to be significant. This result suggests that task orientation may have been promoting athletes’ well-being directly, and not through the associated emphasis on autonomous goal motives. This point is discussed in more detail below, when the results with respect to the fifth hypothesis are analyzed.

The fourth hypothesis was also supported partially. The results confirmed that controlled goal motives were positively associated with physical and emotional exhaustion, as previous studies have shown with other ill-being indicators [28]. However, controlled goal motives were not negatively related to athletes’ vitality, as we hypothesized in the current study. We based this on the theoretical assumption that internal or external pressures would have negative consequences on athletes’ well-being, as some studies have supported [28,32,59]. Nevertheless, previous literature is somewhat equivocal in how this type of goal motive and well-being are related, and in some instances, no relationship has been found between controlled goal motives with indicators of well-being [34] or other adaptive outcomes [42,60,61]. These findings suggest that controlled goal motives lead to higher athletes’ ill-being, while their negative contribution to their well-being or optimal functioning is less clear.

Finally, the fifth hypothesis of this study tested the role of goal motives as mediators in the relationships between goal orientations and well- and ill-being. In this regard, four mediations were analyzed in the path model. The results supported the indirect association between task and ego orientation and physical and emotional exhaustion, both via autonomous and via controlled goal motives. Additionally, ego orientation also contributed to physical and emotional exhaustion via controlled goal motives, but not through autonomous goal motives. An unexpected finding was that the relationships between task and ego orientation with subjective vitality through goal motives were not significant. In fact, only the direct path of task orientation was positively related to athletes’ subjective vitality in our study. As we have commented above, it seems that task orientation and autonomous goal motives shared variance in explaining subjective vitality in our analyses, because once the direct path was added, autonomous goal motives ceased to have a significant relationship with subjective vitality. To further explain these findings, we analyzed the content of the athletes’ self-generated goals, finding that a huge percentage of the sample had personal goals related to improving, learning, or gaining mastery of the task. This goal content clearly reflects task-oriented goals. The fact that the athletes tended to pursue more task-oriented goals may be an artifact of the data for this study being collected at the beginning of the season. The competitive part of the season had not yet started.

This study extended the AGT and SCM research in sport and contributed to understanding that how athletes tend to judge their ability and perceive success relates to the reasons they are pursuing underlying their self-generated goals and reported levels of well- and ill-being. To the best of our knowledge, this is the first study that explored these relationships. Overall, our model suggests that athletes with high levels of task orientation have high autonomous goal motives and low controlled goal motives, which in turn lead to lower levels of physical and emotional exhaustion. Moreover, such athletes also benefit from having high levels of vitality. Conversely, highly ego-oriented athletes pursue their goals with for more highly controlling reasons, which in turn lead to higher levels of physical and emotional exhaustion.

All in all, the present findings provide further evidence for the positive role of task orientation in sport, not only for being a promoter of well-being, but also a preventer of ill-being. In addition, this study provides evidence for the importance of reducing athletes’ ego-orientation, since it may foster experiences of ill-being. Therefore, these results are in consonance with previous studies in sport that have demonstrated the positive association between task orientation with adaptive motivational patterns, in contrast to ego orientation, which has been related with maladaptive ones [11,12,19].

Regarding the study limitations, we must take into account its cross-sectional nature and the limitations of path analysis. Hence, it is not possible to infer causal relationships between the studied variables and, despite the benefits of path analysis to evaluate a priori conceptual models [62], this type of analysis does not control measurement errors. Moreover, the low level of variance explained for the well- and ill-being indicators and the small and moderate bivariate correlations are other limitations. These would indicate that other variables (e.g., motivational climate created by coaches, parents, or peers) could be incorporated into the model in the future to better understand athletes’ subjective vitality and physical and emotional exhaustion. In addition, future studies must consider the possible shared variance between task orientation and autonomous goal motives and take measures to control for it in the analyses. Finally, this study has pointed to the importance of analyzing goal content together with goal motives, in order to understand both what and why, i.e., the reasons and the aims that athletes pursue, as some authors have recommended [26,27]. Qualitative analysis of the self-generated goal content can contribute to a deeper understanding of the results of quantitative analysis, so further research in this line is fundamental.

In spite of these limitations, the results obtained have several implications for the sport domain, emphasizing the importance of the athletes’ dispositional orientations in their goal striving and their experiences of well- and ill-being. The way in which athletes judge success not only could promote or decrease their well- and ill-being, but also could positively contribute to more adaptive or maladaptive goal motives. Specifically, athletes benefit from judging based on self-referenced criteria and avoiding the social comparison to feel more competent. Moreover, this research also pointed out the importance of pursuing the goals with more self-determined motivation, since it is more favorable for athletes in terms of well- and ill-being. Although it has not been analyzed in this study, past literature has shown that coaches, through the creation of motivational climates, could differentially promote task or ego orientation in their athletes [63]. Thus, when coaches create a task involving climate in which they recognize and emphasize exerted effort, cooperation, the mistakes as part of the learning process, and take into account all the contributions that each player makes independently of their ability, they can promote greater task orientation in their athletes [64]. In contrast, if coaches create a strongly ego-involving climate, preferentially recognizing the more talented athletes, punishing mistakes, and promoting rivalry, athletes will be more likely to be highly ego-oriented [65].

Based on the findings of our study, recommendations for coaches would be to engage in more task-involving behaviors and diminish or avoid ego-involving behaviors, in order to help ensure that athletes have more adaptive goal orientations and goal motives, greater subjective vitality, and lower levels of physical and emotional exhaustion. Within the framework of AGT and SDT, an evidence-based program that helps coaches to create these adaptive motivational climates is Empowering Coaching^TM^ [64,66]. This program has been successfully delivered and tested in the sport domain [66,67,68].

## 5. Conclusions

In summary, being highly task-oriented in sport is negatively linked to physical and emotional exhaustion, through the mediation of autonomous and controlled goal motives. In addition, task orientation is positively associated to athletes’ reported subjective vitality. Conversely, ego orientation positively relates to reported physical and emotional exhaustion via the interplay with athletes’ controlled goal motives.

The results found in this study support the positive role of a high task orientation in sport participation. Therefore, in the applied field, it is important that coaches promote task-involving climates, because in this way their athletes can benefit from further optimal functioning, with higher levels of well-being, lower levels of ill-being, and more self-concordant goals.

## Figures and Tables

**Table 1:** Descriptive statistics and reliabilities.

Variables	Range	*M*	*SD*	Alpha	Skewness	Kurtosis
1. Task orientation	1–5	4.47	0.44	0.79	−0.66	−0.09
2. Ego orientation	1–5	2.44	0.87	0.83	0.42	−0.29
3. Autonomous goal motives	1–7	6.33	0.75	0.67	−1.30	1.33
4. Controlled goal motives	1–7	2.36	1.26	0.7	0.57	−0.74
5. Subjective vitality	1–7	4.95	1.1	0.87	−0.29	−0.42
6. Physical and emotional exhaustion	1–5	2.18	0.82	0.89	0.4	−0.35

**Table 2:** Correlations between study variables.

Variables	1	2	3	4	5
1. Task orientation	–				
2. Ego orientation	−0.13 **	–			
3. Autonomous goal motives	0.46 **	−0.04	–		
4. Controlled goal motives	−0.20 **	0.13 **	−0.21 **	–	
5. Subjective vitality	0.25 **	−0.01	0.12 *	−0.05	–
6. Physical and emotional exhaustion	−0.18 **	0.06	−0.22 **	0.26 **	−0.23 **

* *p* < 0.05, ** *p* < 0.01.

## Data Availability

The datasets presented in this article are not readily available because additional studies are undergoing using the dataset. Requests to access the datasets should be directed to natalia.martinez@uv.es.
